# Concomitant malaria among visceral leishmaniasis in-patients from Gedarif and Sennar States, Sudan: a retrospective case-control study

**DOI:** 10.1186/1471-2458-13-332

**Published:** 2013-04-11

**Authors:** Erika van den Bogaart, Marieke MZ Berkhout, Ayman BYM Nour, Pètra F Mens, Al-Badawi A Talha, Emily R Adams, Hashim BM Ahmed, Samira H Abdelrahman, Koert Ritmeijer, Bakri YM Nour, Henk DFH Schallig

**Affiliations:** 1 Department of Biomedical Research, Parasitology Unit, Royal Tropical Institute (KIT), Amsterdam, the Netherlands; 2 Blue Nile National Institute for Communicable Diseases, University of Gezira, Wad Medani, Sudan; 3 Faculty of Medical Laboratory Sciences, University of Gezira, Wad Medani, Sudan; 4 Department of Medicine, Faculty of Medicine, University of Gedarif, and Gedarif Teaching Hospital, Kala Azar Ward, Gedarif, Sudan; 5 Public Health Department, Médecins Sans Frontières, Amsterdam, the Netherlands

**Keywords:** Visceral leishmaniasis, Malaria, Co-infection, Prevalence, Mortality, Risk factors

## Abstract

**Background:**

In areas where visceral leishmaniasis (VL) and malaria are co-endemic, co-infections are common. Clinical implications range from potential diagnostic delay to increased disease-related morbidity, as compared to VL patients. Nevertheless, public awareness of the disease remains limited. In VL-endemic areas with unstable and seasonal malaria, vulnerability to the disease persists through all age-groups, suggesting that in these populations, malaria may easily co-occur with VL, with potentially severe clinical effects.

**Methods:**

A retrospective case-control study was performed using medical records of VL patients admitted to Tabarakallah and Gedarif Teaching Hospitals (Gedarif State) and Al`Azaza kala-azar Clinic (Sennar State), Sudan (2005-2010). Patients positively diagnosed with VL and malaria were identified as cases, and VL patients without microscopy-detectable malaria as controls. Associations between patient characteristics and the occurrence of the co-infection were investigated using logistic regression analysis. Confirmation of epidemiological outcomes was obtained with an independently collected dataset, composed by Médecins Sans Frontières (MSF) at Um-el-Kher and Kassab Hospitals, Gedarif State (1998).

**Results:**

The prevalence of malaria co-infection among VL surveyed patients ranged from 3.8 to 60.8%, with a median of 26.2%. Co-infected patients presented at hospital with deteriorated clinical pictures. Emaciation (Odds Ratio (OR): 2.46; 95% Confidence Interval (95% CI): 1.72-3.50), jaundice (OR: 2.52; 95% CI: 1.04-6.09) and moderate anemia (OR: 1.58; 95% CI: 1.10-2.28) were found to be positively associated with the co-infection, while severity of splenomegaly (OR: 0.53; 95% CI: 0.35-0.81) and, to a less extent, hepatomegaly (OR: 0.52; 95% CI: 0.27-1.01) appeared to be reduced by concomitant VL and malaria. The in-hospital case-fatality rates did not significantly differ between co- and mono-infected patients (OR: 1.13; 95% CI: 0.59-2.17). Conversely, a significantly increased mortality rate (OR: 4.38; 95% CI: 1.83-10.48) was observed by MSF amongst co-infected patients enrolled at Um-el-Kher and Kassab Hospitals, who also suffered an enhanced risk of severe anemia (OR: 3.44; 95% CI: 1.68-7.02) compared to VL mono-infections.

**Conclusions:**

In endemic VL areas with unstable seasonal malaria, like eastern Sudan, VL patients are highly exposed to the risk of developing concomitant malaria. Prompt diagnosis and effective treatment of malaria are essential to ensure that its co-infection does not result into poor prognoses.

## Background

In areas where malaria is co-endemic with visceral leishmaniasis (VL), co-infections with both diseases are common [1]. Previous observations performed through cohorts of VL patients report the occurrence of this co-infection across major VL hot spots [2-5], with prevalence ranging from 31% in Sudan [6] to 1.2% in Bangladesh [7]. At Amudat Hospital, Uganda, where nearly one out of five VL-confirmed patients hospitalized between 2000 and 2006 was co-diagnosed with malaria, concomitant malaria was shown to exacerbate symptoms of VL patients, though with no implications for their prognosis [1]. Children under 10 years of age, notoriously more vulnerable to both malaria and VL, exhibited a twofold higher risk of being co-infected compared to adults. The clinical relevance of the VL-malaria co-morbidity, as attested by its frequency and severity at Amudat Hospital, highlights the risks associated with disease co-endemicity and suggests that other malaria- and VL-endemic areas may experience similar co-infection burdens.

Of the 30,000 to 50,000 annual VL cases estimated in East Africa, the second largest VL focus after South-Asia, half as many (15,700 to 30,300) are thought to occur in Sudan [8,9]. Hyper-endemic foci are located in the eastern part of the country, stretching from the White Nile in the west to the Ethiopian border in the east, and from Kassala State in the north across the border of Southern Sudan up to Upper Nile State in the south [10]. Here, the disease has been reported since the early 1900s [11-13], but it is in the past 30 years that it has reached dramatic proportions, resulting in recurrent epidemics which have claimed hundreds of thousands of lives [14-16]. Currently, Gedarif State in Eastern State, together with the region of Sennar and Singa in Central State, accounts for the vast majority of VL cases in Sudan [17]. The distribution of the disease within these areas is wide, erratic and variable, with highly endemic clusters situated near the banks of the Blue Nile River and its tributaries (Dinder, Rahad and Atbara Rivers) and the Dinder National Park [18-22]. Here, during the outbreaks that cyclically recur (every 7-10 years), the disease can reach incidences of >50 cases per 1000 per year [22]. Diagnostics and treatment services are available at several governmental hospitals and rural dispensaries. However, due to the high cost of treatment, many cases have been referred to Médecins sans Frontières-Holland (MSF-H), whose activities at Um-el-Kher and Kassab VL treatment centers have resulted in more than 24,000 VL patients treated over the last hyper-endemic period (1996-2003) [17]. In the early 2000s, after an initial decline in the disease incidence, a new upsurge in the number of VL cases was recorded [9], leading to the establishment in January 2010 of a new VL ward (MSF-Switzerland) at Tabarakallah Hospital.

Unstable seasonal malaria, due to *Plasmodium falciparum*, prevails in most areas of Gedarif and Sennar States, where prevalence rates of 1.6% and 1.1%, respectively, were confirmed among the communities surveyed in 2009 [23]. This epidemiological pattern of malaria transmission considerably affects the age-related ability of local individuals to acquire immunity against clinical malaria, resulting into seasonal outbreaks that strike all age-groups, albeit with different incidences [24,25]. Subclinical infections are often harbored throughout the dry season, resulting into semi-immunity which ultimately develops within 2 to 3 decades [26]. Concomitant exposure of these individuals to *Leishmania* parasites may, therefore, have dramatic consequences on ability to control both infections, potentially resulting in an aggravated clinical picture.

To describe the clinical impact of VL-malaria co-infections in patients with low malaria immunity, we investigated the epidemiology of VL-malaria co-infections in a VL endemic area characterized by unstable seasonal malaria. By surveying three strategically located hospitals, we obtained a semi-representative data collection of Gedarif and Sennar States (2005-2010), whereby the risk for local VL patients of acquiring a malaria co-infection was assessed. The high prevalence of this co-morbidity and its negative impact on patients’ clinical condition, as observed in this study, highlighted the existence of a clinically significant condition, whose life-threatening implication was indirectly confirmed by an independently collected dataset, gathered by MSF-H at Um-el-Kher and Kassab Hospitals, Gedarif State, in 1998.

## Methods

### Study area

**Figure 1:**
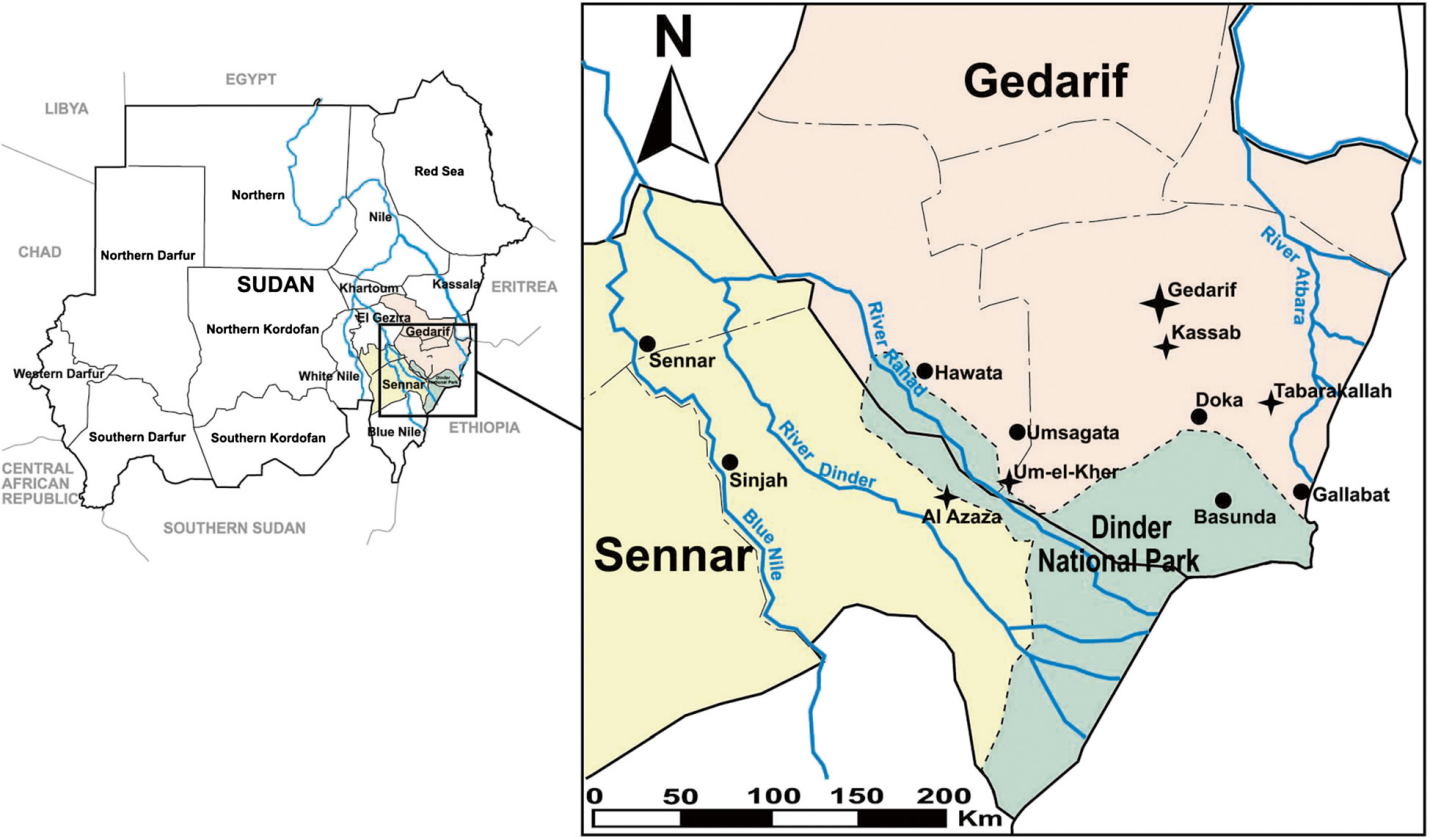
**Map of Gedarif and Sennar States, Sudan, indicating the study sites and neighboring villages.** The study hospitals are shown with a star, while circles indicate neighboring cities and villages.

The study area lies in Sudan, between the Blue Nile River in the central region of Sennar State and the lower Atbara River, which borders Gedarif State in the northeast. The Rahad River and the Dinder River, both tributaries of the Blue Nile River, flow across the region with seasonal regime, marked by major floods during the rainy season (from June to October) and long periods of dry-off throughout the rest of the year. Al`Azaza kala-azar Clinic (Sennar State), Gedarif Teaching Hospital and Tabarakallah Hospital (Gedarif State) (Figure 1) were selected as treatment centers for conducting the survey. The hospitals encompass a VL-dedicated ward, where diagnosis and treatment for VL are performed. Gedarif Teaching Hospital, situated in the city of Gedarif, receives patients from the town and surrounding areas. In addition, difficult clinical cases encountered at regional level are referred to the hospital, which therefore serves as Regional Reference Hospital. Al`Azaza kala-azar clinic, located along the Dinder River, in proximity to the Dinder National Park, is a rural hospital. Together with Gedarif Teaching Hospital, Kassab Hospital and 4 other health centers in Gedarif and Sennar States, the clinic received training and support from MSF-H, as part of a “restructuring program” (2001-2004) for improving local management of VL cases. Tabarakallah Hospital, located in the eastern Atbara region, 20 km south from the Atbara River, is a rural hospital which houses an MSF VL treatment center from January 2010. After departure of MSF-H from the region in 2004, many of the medical staff who worked with MSF in an independent Zakat (Islamic charity)-funded clinic in Tabarakallah, were employed by the hospital.

### Study design and population

A retrospective case-control study was performed on patients positively diagnosed for VL. Patients with a laboratory-confirmed diagnosis of both VL and malaria at hospital admission or during hospitalization were identified as cases, while controls were patients similarly diagnosed with VL, whose blood smears tested negative for malaria. Laboratory-confirmation of VL was obtained either through microscopic examination of lymph node, bone marrow or spleen aspirates, or using serological tests, such as the direct agglutination test (DAT) (titer ≥1:6400) and the rk39 antigen-based dipstick (Kalazar Detect Rapid Test®), in combination with the clinical case definition of the World Health Organization (WHO) [27]. Intensity of *Leishmania* infection in lymph node or bone marrow aspirates was categorized according to the WHO-recommended grading system [28,29]. Briefly, parasitaemias ranging from +1 to +4 were defined as smears containing 1-10 parasites in 1000, 100, 10 or 1 field, respectively, while 10-100 and >100 parasites per field indicated a parasitaemia of +5 and +6, respectively. Malaria was confirmed by microscopic examination of blood smears or by Rapid Diagnostic Tests (RDTs) (SD Bioline Malaria Ag P.f/P.v). Diagnosis of other concomitant diseases was based on clinical suspicion only. Clinical examination of patients included assessment of hemoglobin (Hb) levels (Lovibond method), spleen size (measured in the anterior axillary line to the furthest point during quiet breathing) and liver size (measured in midclavicular line during quiet breathing). Nutritional status, as determined by Weight-for-High percentiles or Body Mass Index (BMI), was directly recorded. Standard treatment for all VL patients, including relapsing cases, consisted of generic sodium stibogluconate administered parenterally. For malaria, artemisinin derivatives (artemether or artesunate), as mono-therapy or in combination with sulfadoxine-pyrimethamine (SP) or lumefantrine, or alternatively quinine were administered as first-line treatment. Test of cure was regularly performed for VL, but not for malaria, by microscopy on lymph node or bone marrow aspirates.

### Data collection

Medical records of patients hospitalized between January 2005 and December 2010 in the three hospitals, were retrospectively reviewed. Data from patients positively diagnosed with VL were collected manually, by using paper Case Record Forms (CRF) specially designed for the study. Data included patients’ demographic details, clinical signs and symptoms, test results, medical treatments and relative outcomes. Upon completion of the collection process, data were single-entered using SPSS 15.0 (SPSS Inc., Chicago, IL, USA) and Microsoft Excel softwares.

### Data analysis

Descriptive analyses were conducted to examine the features of the overall study population and to assess prevalence and case-fatality rates of the co-infected patients. Statistical analysis was performed using SPSS 15.0 software. Characteristics of cases and controls were individually compared, using the Pearson Chi-square test or the Fisher Exact Probability test. Continuous variables were categorized in predefined groups: age (<5, 5-9, 10-19, 20-29 and ≥30 years) based on malaria risk, spleen size (0-3 cm, 4-5 cm and ≥6 cm) by percentiles and anemia based on hemoglobin level (no-mild ≥7.3 g/dl, moderate 5.3-7.2 g/dl and severe <5,3 g/dl). As only few patients (*n* = 22, 2.0%) were found to be non-anemic (Hb ≥11 g/dl), while the majority (n = 606, 55.1%) had mild anemia (Hb 7.3-10.9 g/dl), the groups with no and mild anemic patients were combined in one category, more truly representative of the patient set for anemia risk assessment. Seasonality of the co-infection was examined by categorizing hospital admissions during wet season (from June to October) or dry season (from November to May). Odds ratio (OR) with 95% confidence intervals (CI) were calculated to test whether a variable significantly differed between cases and controls. Multivariable logistic regression models were used to identify independent patients’ characteristics associated with the co-infection. All variables with a *P*-value <0.10 in the univariate analyses were entered stepwise in the multivariate analysis, whose final model only comprised significant variables (*P*-value <0.05) and variables which significantly increased the model fit, as assessed by the −2 Log Likelihood test.

### MSF’s dataset

Demographic and clinical data of VL patients enrolled in a clinical trial conducted by MSF-H at Um-el-Kher and Kassab Hospitals (1998) were analyzed for comparison with the survey outcomes. Both hospitals lie in Gedarif State (Figure 1), in proximity to the Rahad River and the Gedarif town, respectively, where prevalence of malaria and VL averages the rates characterizing the survey sites. The trial conducted by MSF aimed to compare efficacy and safety of branded *versus* generic sodium stibogluconate for the treatment of primary VL. Hence, the study population comprised patients with a laboratory-confirmed diagnosis of VL (DAT titer ≥1:6400 or demonstration of *Leishmania* on aspirates of spleen or lymph node) and no history of previous anti-leishmanial treatment. Microscopic search for malaria was also performed on all study subjects at enrollment. Treatment for VL consisted of either Pentostam or generic sodium stibogluconate given intramuscularly (20 mg/kg/day for 30 days), while SP and quinine were administered as first-line treatment for uncomplicated and severe malaria, respectively. Using the same case-control definition as in the survey, descriptive and statistical analyses (Person Chi-square test) were performed to assess prevalence and mortality rates of the VL-malaria co-infection and to measure its association with explanatory variables.

### Ethical approval

Data were collected as part of routine patient care, with no need for additional investigations. Data extraction from medical records was performed by anonymizing the information recorded in the CRFs. Ethical approval for the study was obtained from the Health Directorates of Gedarif and Sennar States (3^rd^ January 2011) and from the Ethics Review Board of MSF (19^th^ July 2012).

## Results

### Prevalence of VL-malaria co-infections in Gedarif and Sennar States (2005-2010)

A total of 1324 medical records reporting on VL-diagnosed patients hospitalized in Gedarif Teaching Hospital, Al`Azaza kala-azar Clinic and Tabarakallah Hospital during the study period were reviewed for the survey. Of these, 1295 reported on patients with a laboratory-confirmed VL infection, who were included in the study, while the remaining 29 were excluded for lack of laboratory diagnostic evidence. Microscopy, mainly performed on lymph node (*n* = 848, 65.5%), bone marrow (*n* = 428, 33.1%) and/or spleen (*n* = 3, 0.2%) aspirates, was used for the diagnosis of VL. Serology was performed in 17 patients, 13 of whom were confirmed by DAT and 4 by rk39 antigen-based dipstick. Overall, 404 (31.2%) of the VL-confirmed patients were co-diagnosed with malaria at hospital admission or during hospitalization. In all cases but one, in which diagnosis was established with an RDT, malaria was confirmed by microscopy. Stratification of the study population by hospital resulted into an unevenly distributed co-infection rate, with a prevalence of 3.8% in Gedarif Teaching Hospital (*n* = 18), followed by Tabarakallah Hospital (*n* = 84) and Al`Azaza kala-azar Clinic (*n* = 302) with 26.2% and 60.8%, respectively. With the exception of one *P. vivax*-infection, all malaria cases for which the *Plasmodium* species was determined, were attributed to *P. falciparum* (*n* = 396, 98.0%).

### Demographic and clinical features of VL-malaria co-infections in Gedarif and Sennar States (2005-2010)

With a median age of 12 years (inter-quartile range 5-23 years), nearly three quarters of VL-malaria co-infected patients were under 20 years old. Stratified by age, the percentage of cases was highest among children aged between 0 and 4 years (40.7%, *n* = 120/296) and progressively reduced until 29 years (27.0%, *n* = 50/185)(data not shown), indicating that the chance of detecting malaria in patients already diagnosed for VL declined as age increased. Residents of Sennar State appeared to be mostly stricken by the co-infection, particularly in the villages of Al`Azaza (28.0%), Om Bagraa (8.7%) and Jaldook (5.2%). Anemia (hemoglobin level <11 g/dl) was a hallmark of nearly all co- and mono-infected patients (91% and 79%, respectively), with most patients being diagnosed with mild or moderate anemia on hospital admission. Co-occurrence of malaria in VL patients resulted in increased severity of the anemia status, as shown by the decrease in median hemoglobin level (7.0 g/dl, inter-quartile range 6.5-8.0 g/dl for cases and 8.0 g/dl, 7.0-9.0 g/dl for controls). Despite most co- and mono-infected patients presented with enlarged spleen, frequency (92% for cases, 95% for controls) and severity of splenomegaly (median spleen size below the costal margin 4.0 cm, inter-quartile range 2.0-6.0 cm for cases and 5.0 cm, 2.0-8.0 cm for controls) appeared to be slightly reduced by concomitant malaria.

In Al`Azaza kala-azar Clinic and Tabarakallah Hospital, most co-infections were detected among young boys (median age 9 and 10 years, inter-quartile range 3-23 years and 4-18 years, respectively) (Table 1). In Gedarif Teaching Hospital, conversely, cases unlike controls mainly consisted of girls (55.6%, *n* = 10/176) (Table 1), although the difference appeared to be statistically not significant. Seasonal distribution of hospital admissions was similar in all the three hospitals, with most patients presenting at hospital during the long dry season (November-May). Co-occurrence of malaria in VL patients resulted in a sharper division between dry and wet season, particularly in Gedarif Teaching Hospital, where 82.4% of all co-infected patients were hospitalized between November and May, emphasizing the peculiar time-trend of these co-infections (in these regions malaria usually occurs during or shortly after the rains, while VL clinical cases peak during the dry season). Finally, with a median disease duration of 12 days from the initial onset of symptoms, co-infected patients presented at Tabarakallah Hospital earlier than at Al`Azaza kala-azar Clinic (21 days) or Gedarif Teaching Hospital (30 days).

**Table 1:** Characteristics of the overall VL population and VL-malaria co-infected patients, stratified by hospital, Sudan (2005-2010)

**Treatment center**	**Gedarif Teaching Hospital**	**Tabarakallah Hospital**			**Al`Azaza Kala-azar Clinic**	
	Total population	Co-infected patients	Total population	Co-infected patients	Total population	Co-infected patients
	*n* (%)	*n* (%)	*n* (%)	*n* (%)	*n* (%)	*n* (%)
**Number of patients**	**468**	**18 (3.8)**	**321**	**84 (26.2)**	**497**	**302 (60.8)**
**Gender**	**456**	**18**	**321**	**84**	**497**	**302**
Male	280 (61.4)	8 (44.4)	179 (55.8)	49 (58.3)	254 (51.1)	155 (51.3)
Female	176 (38.6)	10 (55.6)	142 (44.2)	35 (41.7)	243 (48.9)	147 (48.7)
**Age**	**440**	**15**	**321**	**84**	**492**	**301**
Median (years) (inter-quartile range)	14.0 (7.0-24.5)	13.0 (9.0-29.0)	10.0 (5.0-18.0)	10.0 (4.2-17.7)	9.0 (3.0-23.0)	9.0 (3.0-23.0)
0-4 years	68 (15.5)	2 (13.3)	77 (24.0)	21 (29.8)	151 (30.7)	97 (32.2)
5-9 years	87 (19.8)	3 (20.0)	71 (22.1)	19 **(**22.6)	99 (20.1)	55 (18.3)
10-19 years	127 (28.9)	5 (33.3)	99 (30.8)	28 (33.3)	91 (18.5)	59 (19.6)
20-29 years	89 (20.2)	2 (13.3)	28 (8.7)	6 (7.1)	68 (13.8)	42 (14.0)
≥30 years	69 (15.7)	3 (20.0)	46 (14.3)	10 (11.9)	83 (16.9)	48 (15.9)
**Season**	**452**	**17**	**321**	**84**	**488**	**297**
Wet season	151 (33.4)	3 (17.6)	124 (38.6)	32 (38.1)	226 (46.3)	131 (44.1)
Dry season	301 (66.6)	14 (82.4)	197 (61.4)	52 (61.9)	262 (53.7)	166 (55.9)
**Duration on-going sickness**	**433**	**17**	**302**	**82**	**441**	**259**
Median (days) (inter-quartile range)	30.0 (15.0-40.0)	30.0 (13.5-76.0)	14.0 (9.0-21.0)	12.0 (7.0-14.0)	30.0 (14.0-61.0)	21.0 (14.0-61.0)

### Infection intensity and case-fatality rate of VL-malaria co-infections in Gedarif and Sennar States (2005-2010)

Intensity of VL infection, as determined by parasitaemia in lymph node or bone marrow aspirates, was directly related with the frequency of VL-malaria co-infections. Stratified by infection intensity, in fact, the ratio of VL-malaria co-infections was lowest in the groups with less severe VL (*n* = 339/757, 44.8% as determined in lymph node aspirates and *n* = 15/259, 5.8% as determined in bone marrow aspirates) and gradually increased as VL infection intensified (*n* = 3/4, 75.0% and *n* = 1/5, 20.0% in lymph node and bone marrow aspirates, respectively, with the highest intensity) (Table 2).

**Table 2:** VL-malaria co-infections, stratified by VL infection intensity, and its association with the overall in-hospital death, Sudan (2005-2010)

**Infection intensity of VL in aspirates**	***n *cases/ denominators**	**%**	**In-hospital deaths *n *(%)**	**Discharged alive *n *(%)**	**Odds Ratio**	**95% Confidence Interval**
**lymph node**	**377/824**		**21**	**790**		
+1	339/757	44.8	18 (85.7)	727 (92.0)	1	
+2	24/44	54.5	2 (9.5)	41 (5.2)	1.97	0.44-8.78
+3	11/19	57.9	0	19 (2.4)	1.00	0.99-1.00
+4	3/4	75.0	1 (4.8)	3 (0.4)	13.46	1.34-135.77*
**bone marrow**	**16/264**		**13**	**247**		
+1	15/259	5.8	11 (84.6)	244 (98.8)	1	
+2	1/5	20.0	2 (15.4)	3 (1.2)	14.79	2.24-97.74*

*P-value <0.10 based on Fisher Exact Probability tests.

During hospitalization, 14 co-infected patients died, resulting in an overall case-mortality rate of 3.5%. A similar case-fatality rate was found among the controls (3.1%, *n* = 27) (OR: 1.13; 95% CI: 0.59-2.17), indicating that concomitant malaria did not represent a risk factor for poor prognosis. When stratified by malaria treatment, in-hospital fatalities clustered amongst co-infected patients treated with artemether and quinine, whose mortality risk was 13-fold (OR: 13.34; 95% CI: 0.77-229.85) and 15-fold (OR: 15.35; 95% CI: 0.81-289.33) higher, respectively, than in patients receiving artesunate+SP (Table 3). Patients with a high density of *Leishmania* parasites were overrepresented among those who died, confirming previous findings, whereby high intensities of VL infection increased the mortality risk [30]. Here, patients harboring high numbers of *Leishmania* parasites in their lymph node or bone marrow were found to be 13 (OR: 13.46; 95% CI: 1.34-135.77) and 15 (OR: 14.79; 95% CI: 2.24-97.74) times more likely to die, respectively (Table 2).

**Table 3:** VL-malaria co-infections, stratified by malaria treatment, and their association with in-hospital death, Sudan (2005-2010)

**Malaria treatment**	***n *in-hospital fatalities / denominator**	**%**	**Odds Ratio**	**95% Confidence Interval**
Artesunate + SP	0/118	0	1	
Artemether	10/186	5.4	13.34	0.77-229.85*
Quinine	4/69	5.8	15.35	0.81-289.33*
Artesunate	0/15	0	-	-
Artemether + lumefantrine	0/9	0	-	-

*P-value <0.10 based on Fisher Exact Probability tests. For the statistical analysis, a value of 0.5 was added to each variable to ensure calculation of OR and 95% CI.

### Risk factors for VL-malaria co-infections in Gedarif and Sennar States (2005-2010)

Associations between demographic and clinical variables and the co-infection, as described by univariate analysis, are summarized in Table 4. Gender and age were identified as risk factors for the VL-malaria co-infection. Significant associations were also found to link the co-infected patients with the treatment center in which they have been hospitalized. Neither the rainy season nor the intake of anti-malarial drugs prior to hospitalization significantly altered the risk for VL patients of acquiring malaria. Malnourishment was relatively more common among cases, resulting in a positive association between severe malnutrition and the co-infection (OR: 2.21; 95% CI: 1.01-4.85). A similar positive association was found with moderately (OR: 1.73; 95% CI: 1.32-2.28) or severely anemic patients (OR: 1.50; 95% CI: 0.99-2.28). The likelihood of being diagnosed with concomitant VL and malaria decreased as spleen size increased: patients with massive splenomegaly (spleen size ≥6 cm below the costal margin) were found to be more than twice less likely to be co-infected (OR: 0.38; 95% CI: 0.26-0.55) compared with patients with no or minor spleen enlargement. In some patients, malaria displayed an exacerbating, although not significant, effect on VL infection, as revealed by the increased number of *Leishmania* parasites observed in their lymph node or bone marrow aspirates. Finally, while hepatomegaly was less frequently observed in co-infected patients compared to mono-infected patients (OR: 0.29; 95% CI: 0.18-0.48), jaundice (OR: 2.85; 95% CI: 1.43-5.66) and, particularly, weight loss (OR: 3.04; 95% CI: 2.37-3.90) were more commonly reported among co-infected cases.

**Table 4:** Univariate analysis of characteristics associated with VL-malaria co-infections, Sudan (2005-2010)

**Variable**	**Cases *n *(%)**	**Controls *n *(%)**	**Crude Odds Ratio**	**95% Confidence Interval**
**Gender**	**404**	**870**		
Male	212 (52.5)	501 (57.6)	1	
Female	192 (47.5)	369 (42.4)	1.23	0.97-1.56*
**Age**	**400**	**853**		
<5 years	120 (30.0)	176 (20.6)	1.53	1.05-2.24*
5-9 years	77 (19.3)	180 (21.1)	0.96	0.64-1.44
10-19 years	92 (23.0)	225 (26.4)	0.92	0.62-1.35
20-29 years	50 (12.5)	135 (15.8)	0.83	0.53-1.30
≥30 years	61 (15.3)	137 (16.1)	1	
**Treatment center**	**404**	**882**		
Gedarif Teaching Hospital	18 (4.4)	450 (51.0)	1	
Tabarakallah Hospital	84 (20.8)	237 (26.9)	8.86	5.20-15.10*
Al`Azaza kala-azar Clinic	302 (74.8)	195 (22.1)	38.72	23.38-64.11*
**Season**	**398**	**863**		
Dry season	232 (58.3)	528 (61.2)	1	
Wet season	166 (41.7)	335 (38.8)	1.13	0.89-1.44
**Previous anti-leishmanial treatment**	**389**	**860**		
No	373 (95.9)	806 (93.7)	1	
Yes	16 (4.1)	54 (6.3)	0.64	0.36-1.13
**Previous anti-malarial treatment**	**396**	**864**		
No	277 (70.0)	604 (69.9)	1	
Yes	119 (30.0)	260 (30.1)	1.00	0.77-1.29
**Malnutrition**	**322**	**575**		
None	262 (81.4)	497 (86.4)	1	
Mild	17 (5.3)	25 (4.3)	1.29	0.68-2.43
Moderate	29 (9.0)	41 (7.1)	1.34	0.82-2.21
Severe	14 (4.4)	12 (2.1)	2.21	1.01-4.85*
**Anemia degree on admission**	**374**	**725**		
Median Hb (inter-quartile range)	7.0 (6.5-8.0)	8.0 (7.0-9.0)		
None-mild (Hb ≥7.3 g/dl)	182 (48.7)	445 (61.4)	1	
Moderate (Hb 5.3-7.2 g/dl)	149 (39.8)	210 (29.0)	1.73	1.32-2.28*
Severe (Hb <5.3 g/dl)	43 (11.5)	70 (9.7)	1.50	0.99-2.28*
**Spleen size**	**238**	**563**		
Median (inter-quartile range)	4.0 (2.0-6.0)	5.0 (2.0-8.0)		
0-3 cm	94 (39.5)	150 (26.6)	1	
4-5 cm	79 (33.2)	141 (25.0)	0.89	0.61-1.30
≥6 cm	65 (27.3)	272 (48.3)	0.38	0.26-0.55*
**Infection intensity in lymph node aspirate**	**377**	**447**		
+1	339 (89.9)	418 (93.5)	1	
+2	24 (6.4)	20 (4.5)	1.48	0.80-2.72
+3	11 (2.9)	8 (1.8)	1.70	0.67-4.26
+4	3 (0.8)	1 (0.2)	3.70	0.38-35.72
**Infection intensity in bone marrow aspirate**	**16**	**248**		
+1	15 (93.8)	244 (98.4)	1	
+2	1 (6.2)	4 (1.6)	4.07	0.43-38.68
**Symptoms**	**404**	**880**		
Hepatomegaly	19 (4.7)	128 (14.5)	0.29	0.18-0.48*
Jaundice	19 (4.7)	15 (1.7)	2.85	1.43-5.66*
Weight loss	278 (68.8)	370 (42.0)	3.04	2.37-3.90*

*P-value <0.10 based on Person Chi-square tests

All 11 variables associated with the VL-malaria co-infection in the univariate analysis were included in the multivariable models. Due to the high number of missing data in reporting clinical signs and symptoms, three different models were designed to explore the association between the selected variables and the VL-malaria co-infection: model A, in which the independent risk factors were identified, model B describing the association between clinical signs and symptoms and the co-infection and model C, which combines all variables (risk factors, clinical signs and symptoms) in one analysis. Model A (Table 5) shows that, after adjusting for the other variables included in the model, the treatment center in which co-infected patients were hospitalized, and therefore its catchment area, remained the most important factor associated with the risk of being co-infected. After adjusting for the other variables included in the model, gender and age lost their statistical significance. The variable age, however, was kept in the model as it significantly increased its fit.

**Table 5:** Model A: Multivariate analysis of risk factors for VL-malaria co-infections among VL patients, Sudan (2005-2010)

***n *= 1253**	**Odds Ratio**	**95% Confidence Interval**	***P*-value**
**Treatment center**			
Gedarif Teaching Hospital	1	-	<0.001
Tabarakallah Hospital	9.88	5.57-17.55	<0.001
Al`Azaza kala-azar Clinic	44.85	25.89-77.71	<0.001
**Age**			
<5 years	1.26	0.81-1.97	0.305
5-9 years	1.01	0.63-1.62	0.972
10-19 years	1.31	0.83-2.09	0.246
20-29 years	1.06	0.62-1.80	0.842
≥30 years	1	-	0.608

In model B (Table 6), logistic regression was performed on the sub-cohort of patients for whom symptoms, besides other features, were reported. After adjusting for the other variables included in the model, malnutrition lost its statistical significance and was therefore excluded from the model. The variable hepatomegaly also lost most of its significance, but a slightly negative association could still be recognized (OR: 0.52; 95% CI: 0.26-1.01). The risk for VL patients of developing jaundice (OR: 2.52; 95% CI: 1.04-6.09) or undergoing weight loss (OR: 2.46; 95% CI: 1.72-3.50), on the other hand, was doubled by the concomitancy of malaria.

**Table 6:** Model B: Multivariate analysis of clinical signs and symptoms associated to VL-malaria co-infections, Sudan (2005-2010)

***n *= 690**	**Odds Ratio**	**95% Confidence Interval**	***P*-value**
**Symptoms**			
Hepatomegaly	0.52	0.26-1.01	0.052
Jaundice	2.52	1.04-6.09	0.041
Weight loss	2.46	1.72-3.50	<0.001
**Spleen size**			
0-3 cm	1	-	0.007
4-5 cm	0.90	0.60-1.36	0.629
≥6 cm	0.53	0.35-0.81	0.003
**Anemia**			
None - mild (Hb ≥7.3 g/dl)	1	-	0.440
Moderate (Hb 5.3-7.2 g/dl)	1.58	1.10-2.28	0.013
Severe (Hb <5.3 g/dl)	1.10	0.63-1.93	0.737

Massive splenomegaly was less common among co-infected patients (OR: 0.53; 95% CI: 0.35-0.81), who instead suffered frequent exacerbations of their anemic status, as observed in the group with moderate anemia (OR: 1.58; 95% CI: 1.10-2.28).

Combining all variables in one model (model C, Table 7) resulted in the loss of significance for all variables, except the one involving the treatment center, which possibly displayed a confounding effect on all other associations. The model confirmed a key role for this variable, which led to an 8- and 42- fold different likelihood of being co-diagnosed with VL and malaria in Tabarakallah Hospital (OR: 8.02; 95% CI: 3.68-17.46) and Al`Azaza kala-azar Clinic (OR: 41.66; 95% CI: 20.01-86.72), respectively, compared to Gedarif Teaching Hospital.

**Table 7:** Model C: Multivariate analysis of risk factors, clinical signs and symptoms associated to VL-malaria co-infections, Sudan (2005-2010)

***n *= 690**	**Odds Ratio**	**95% Confidence Interval**	***P*-value**
**Treatment center**			
Gedarif Teaching Hospital	1	-	<0.001
Tabarakallah Hospital	8.02	3.68-17.46	<0.001
Al`Azaza kala-azar Clinic	41.66	20.01-86.72	<0.001
**Spleen size**			
0-3 cm B.C.M.	1	-	0.706
4-5 cm B.C.M.	1.20	0.76-1.90	0.428
≥6 cm B.C.M.	1.15	0.71-1.86	0.563
**Anemia**			
None - mild (Hb ≥7.3 g/dl)	1	-	0.219
Moderate (Hb 5.3-7.2 g/dl)	1.28	0.85-1.94	0.239
Severe (Hb <5.3 g/dl)	0.74	0.39-1.40	0.356

### MSF’s dataset

Between November and December 1998, 516 primary-VL cases were diagnosed in the MSF kala-azar treatment centers of Um-el-Kher and Kassab. Positive DAT results were obtained for most patients (*n* = 440, 86%), whose diagnosis was occasionally confirmed, as for all patients with borderline DAT titers (from 1:400 to 1:6400), by parasitological evidence in lymph node or spleen aspirates (*n* = 140, 27%). Microscopy-confirmed malaria was co-diagnosed in 89 VL patients (17.2%), 10 of whom died (11.2%). Given the lower case-fatality rate among controls (2.8%), here co-infections significantly increased the mortality risk of VL patients by nearly four and half fold (OR: 4.38; 95% CI: 1.83-10.48) (Table 8). Of the 10 co-infection-related fatalities, 4 were considered to be caused by anemia, and one by cerebral malaria. Although no clear differences in the median hemoglobin level of VL-malaria co-infected patients (7.5 g/dl) *versus* the mono-infected VL patients (8.2 g/dl) could be detected, the proportion of patients with severe anemia (Hb <5.3 g/dl) was significantly higher among cases (15.7%) than among controls (5.2%) (OR: 3.44; 95% CI: 1.68-7.02). No other major differences in nutritional status, spleen size below the costal margin and duration of on-going disease prior to hospitalization were observed between co- and mono-infected patients, except their distribution between the two treatment centers. With more than 80% of the co-infection cases recorded in Um-el-Kher Hospital, the risk here, of being co-diagnosed with VL and malaria was nearly double (OR: 1.94; 95% CI: 1.10-3.41) than at Kassab Hospital.

**Table 8:** **Characteristics of the malarial VL co-infected patients *versus *the non-malarial VL patients enrolled in the MSF’s clinical trial at Um-el-Kher and Kassab Hospitals, Sudan (1998)**

**Variable**	**Cases *n *(%)**	**Controls *n *(%)**	**Crude Odds Ratio**	**95% Confidence Interval**
**Age**	**89**	**427**		
Median (years), (interquartile range)	7 (4-14)	10 (6-17)		
**Treatment center**	**89**	**427**		
Kassab Hospital	17 (19.1)	134 (31.4)	1	
Um-el-Kher Hospital	72 (80.9)	293 (68.6)	1.94	1.10-3.41*
**Fatal outcome**	**89**	**427**		
No	79 (88.8)	415 (97.2)	1	
Yes	10 (11.2)	12 (2.8)	4.38	1.83-10.48*
**Hb level**	**89**	**427**		
Median (g/dl), (interquartile range)	7.5 (6.0-9.0)	8.2 (7.0-9.4)		
≥5.3 g/dl	75 (84.3)	405 (94.8)	1	
<5.3 g/dl	14 (15.7)	22 (5.2)	3.44	1.68-7.02*
**Nutritional status**	**68**	**299**		
Mean Weigh for Height (%)	78.8	80.4		
Weight for Height Percent ≥70%	60 (88.2)	279 (93.3)	1	
Weight for Height Percent <70%	8 (11.8)	20 (6.7)	1.86	0.78-4.42
**Spleen size below the costal margin**	**80**	**389**		
Median (cm), (interquartile range)	6 (3-8)	7 (4-10)		
<6 cm	33 (41.2)	144 (37.0)	1	
≥6 cm	47 (58.8)	245 (63.0)	0.84	0.51-1.37
**Duration on-going sickness**	**86**	**422**		
Median (days), (interquartile range)	30 (15-60)	30 (20-60)		
<60 days	62 (72.1)	286 (67.8)	1	
≥60 days	24 (27.9)	136 (32.2)	0.81	0.49-1.36

*P-value <0.05 based on Person Chi-square test.

## Discussion

This is the first multicenter retrospective survey ever undertaken in the field of VL-malaria co-infections. The study describes the epidemiology of concomitant malaria among VL in-patients from Gedarif Teaching Hospital, Tabarakallah Hospital and Al`Azaza kala-azar Clinic, eastern Sudan (2005-2010) and confirms its clinical relevance by comparing prevalence and mortality rates with an antecedent (1998), independently collected dataset from the same region (Um-el-Kher and Kassab Hospitals). Not only the risk of co-acquiring VL and malaria appears to be substantial in these areas, with a significant geographical variation, but the clinical implications deriving from being co-infected provide the evidence for a public health concern. Exacerbated clinical pictures and increased mortality risk, possibly due to inadequate antimalarial treatment, were highlighted by this survey, suggesting that prompt diagnosis and effective therapy of concomitant malaria is needed to ensure positive resolution of the VL-malaria co-infection.

Ranging from 3.8 to 60.8% and with a median of 26.2%, the prevalence of malaria co-infection among VL surveyed patients (2005-2010) confirms the frequent superimposing of the two diseases in rural areas of Gedarif and Sennar States. Although these estimates may be higher than expected, based on the local malaria transmission rates, similar figures have been found in the MSF’s dataset from Um-el-Kher and Kassab Hospitals, where 19.7% and 11.3%, respectively, of the VL patients enrolled in the clinical trial (1998) were positive for malaria. Again at Um-el-Kher Hospital, clinical studies conducted between January 2004 and early 2005 revealed that 15% of pregnant VL women enrolled in the trial [31] and 31% of Ambisome-treated VL patients [6] were co-diagnosed with malaria, while a 4.8% rate was found in Kassab (2005-2006) [32], where the malaria prevalence is notoriously lower. In agreement with previous observations performed at Amudat Hospital, Uganda (2000-2006) [1], where a co-infection rate of 19% was reported using the same criteria as here, the frequent co-occurrence of malaria in VL patients suggests that these patients may have increased susceptibility towards the malarial infection, possibly due to a VL-promoted impairment of the immune system. The clustering of most co-infection diagnoses in Al`Azaza kala-azar Clinic (74.8%), however, followed by Tabarakallah Hospital (20.8%) and Gedarif Teaching Hospital (4.4%) is rather unexpected. In Gedarif Teaching Hospital, only 3.8% of the VL-confirmed patients were co-diagnosed with malaria, a figure which may be explained by its function as Regional Reference Hospital, besides the low malaria burden found in this urban area. Difficult cases encountered in rural hospitals and referred to Gedarif Teaching Hospital, usually received, prior to admission to the Regional Hospital, a full course of anti-malarial treatment to exclude malaria as a possible complication. This may therefore have resulted in a lower percentage of VL patients having malaria on hospital admission. The longer disease duration described among VL patients hospitalized in Gedarif Teaching Hospital compared to the other two study sites, and their villages of origin, seem to confirm that a large number of these patients may not have presented to this hospital as a first-line action. If, therefore, a higher malaria-VL co-infection rate is to be expected in rural hospitals of Gedarif and Sennar State, the figure obtained in Al`Azaza kala-azar Clinic (60.8%), appears to somehow overestimate the burden posed by this co-morbidity. Given that higher malaria rates, favored by the proximity with the river and natural reserve, might have locally occurred, poor quality of malaria diagnosis in Al`Azaza kala-azar Clinic cannot be excluded.

Concomitant malaria partly exacerbated the clinical picture of VL patients, who presented with more frequent emaciation, icterus and moderate anemia. Two scenarios may be postulated: co-infected patients may either have suffered from malaria-associated symptoms which, in addition to the VL ones, have caused deterioration of their clinical condition and/or have run an exacerbated course of VL due to concomitant malaria, in which case symptoms may be related to VL rather than to malaria. If this latter hypothesis may find its rationale in the increased number of *Leishmania* parasites observed in aspirates of co-infected patients, the first speculation may be supported by the peculiar symptom pattern. Jaundice, in fact, is rarely described among VL patients, while it is not uncommon in *P. falciparum* malaria [33]. Weight loss and anemia, on the other hand, are hallmark of both VL and malaria and an increased severity of the anemic status, as observed in co-infected patients, may therefore be the result of an added effect displayed by both diseases on the polyparasitized host. In apparent contradiction is the finding, whereby co-infected patients suffered from less severe hepato-splenomegaly. Suggesting a less advanced state of the diseases in the co-infected patients, the result may be explained by their earlier hospitalization compared to mono-infected VL patients. Patients with concomitant VL and malaria, in fact, presented at hospital nearly 10 days earlier, on average, than those with only VL, possibly due to their more severe symptomatology. Importantly, this may have also had positive implications for their prognosis, which was found to be similar to the controls’ one.

In antithesis to the positive resolution of VL-malaria co-infections during the 2005-2010 survey, is the significantly higher fatality rate (*P*-value 0.001) associated with co-infected patients enrolled by MSF at Um-el-Kher and Kassab Hospital in 1998. During this trial, co-infected patients were nearly four and half times more likely to die compared with the VL mono-infected patients, whose mortality (2.8%) on the other hand, compares well to what found in the most recent survey (3.1%). Different anti-malarial regimen were used within the two study groups: SP and quinine in 1998 for uncomplicated and severe malaria, respectively; artemisinin derivatives (alone or in combination) and more rarely quinine between 2005 and 2010. Sudan’s choice to introduce artemisinin-based combination therapies (ACTs) for treatment of uncomplicated and severe malaria was implemented nation-wide in 2004 [34], following increasing evidence of resistance against chloroquine, SP and quinine, for which failure rates up to 76.9, 16.1 and 16.7%, respectively, were recorded in eastern Sudan prior to ACT era [35-38]. The 11.2% mortality rate of VL-malaria co-infections observed in 1998 at Um-el-Kher Hospital may therefore have partially resulted from treatment failures attributable to either SP or quinine, besides the more severe malaria course in patients receiving quinine. In fact, no major differences for age, median Hb level, nutritional status, spleen size and duration of on-going disease distinguished the co-infected patients’ group at Um-el-Kher and Kassab Hospitals from the one surveyed in 2005-2010 and from its relative controls. The only exception is to be found in the significantly higher number of VL patients who developed severe anemia when co-infected with malaria, similarly to what was observed during the 2005-2010 survey, though to a less extent. Hence, concomitant malaria may not only cause aggravation of VL patients’ clinical condition, but it may also result in a poorer prognosis, if failed to be treated. Among co-infected patients surveyed in 2005-2010, an increased mortality risk, not ascribable to differences in *Leishmania* intensities, was observed when quinine (*P*-value 0.07) and artemether (*P*-value 0.04) were administered as antimalarials, suggesting either increased malaria severity or inadequate drug treatment.

The population surveyed within this study consists of VL patients residing in over 300 different villages, mainly located in Gedarif and Sennar States. Within these districts, Tabarakallah Hospital and Al`Azaza kala-azar Clinic are two rural hospitals receiving patients from some of the worst-affected villages. It should be noted, however, that the cohort of VL patients surveyed here might be sub-representative of the local VL community, as the number of VL-related hospitalizations carried out by the two MSF’s treatment centers in Gedarif Sate (>4000 per year between 1997 and 1999 [22]) exceeds by far the one recorded by the three study hospitals (1324 in total between 2005 and 2010). Other limitations apply to this study, the most important ones being the lack of non-VL malaria infected patients and the quality of diagnosis. Unlike VL, uncomplicated malaria infections are commonly treated on an outpatient basis in hospitals, clinics or simple practices, resulting in few data being systematically recorded by the different facilities. Moreover, malaria patients are rarely found in VL-dedicated hospitals, such as those surveyed in this study. This resulted into the lack of valid malaria controls, essential to investigate whether VL might predispose or rather protect towards a malarial attack and whether it might influence its course and clinical presentation. In absence of quality control, quality of diagnosis remains questionable. Variable outcomes, as documented in medical records, may have suffered from poor standardization, due to the different techniques implemented by clinicians in the different treatment centers and the possible involvement of different health workers in filling these files.

## Conclusion

Based on the results of this study, we conclude that VL patients living in areas with unstable seasonal malaria, such as eastern Sudan, are highly exposed to the risk of developing concomitant malaria. Large variation in the geographical distribution of co-infection cases highlights the presence of environmental and/or social factors, whose identity and relevance in the risk of co-acquiring VL and malaria still remain to be elucidated. Clinical concerns should arise when the two diseases co-occur in the same patients, as significant exacerbation of their clinical condition was observed, along with an increased mortality risk, possibly associated with inappropriate anti-malarial treatment. Local health care policies should take into account the high co-infection burden borne by VL foci with unstable malaria, by recommending systematic malaria screening for all VL patients and ACTs for treatment of malaria.

## Competing interests

The authors declare that they have no competing interests.

## Authors’ contributions

EvdB contributed in conceiving of the study, drafted the study protocol and the manuscript and participated in the data analysis. MB performed the data entry and the statistical analysis and helped to draft the manuscript. AN performed the collection and entry of data. PM and EA participated in conceiving of the study and reviewed the study protocol and the manuscript. AT participated in the collection and entry of data and helped to draft the study protocol. HA participated in the collection and entry of data. SA reviewed the study protocol and the manuscript. KR participated in the data analysis and reviewed the manuscript. BN coordinated the collection and entry of data, organized local logistics, and helped to review the study protocol and the manuscript. HS conceived the study and participated in its design, and reviewed the study protocol and the manuscript. All authors read and approved the final manuscript.

## Pre-publication history

The pre-publication history for this paper can be accessed here:

http://www.biomedcentral.com/1471-2458/13/332/prepub
